# Subtotal Nephrectomy Associated with a High-Phosphate Diet in Rats Mimics the Development of Calcified Aortic Valve Disease Associated with Chronic Renal Failure

**DOI:** 10.3390/jcm12041539

**Published:** 2023-02-15

**Authors:** Hind Messaoudi, Thomas Levesque, Nicolas Perzo, Elodie Berg, Guillaume Feugray, Anaïs Dumesnil, Valéry Brunel, Dominique Guerrot, Hélène Eltchaninoff, Vincent Richard, Saïd Kamel, Eric Durand, Youssef Bennis, Jérémy Bellien

**Affiliations:** 1 INSERM EnVI UMR 1096, University of Rouen Normandie, F-76000 Rouen, France; 2 Department of Cardiology, CHU Rouen, F-76000 Rouen, France; 3 Department of Thoracic Surgery, CHU Rouen, F-76000 Rouen, France; 4 Department of General Biochemistry, CHU Rouen, F-76000 Rouen, France; 5 Department of Nephrology, CHU Rouen, F-76000 Rouen, France; 6 Department of Pharmacology, CHU Rouen, F-76000 Rouen, France; 7 UR UPJV 7517, Mécanismes Physiopathologiques et Conséquences des Calcifications Cardiovasculaires (MP3CV), Centre de Recherche Universitaire en Santé, Université de Picardie Jules Verne, F-80054 Amiens, France; 8 Department of Biochemistry, Amiens-Picardie University Hospital, F-80054 Amiens, France; 9 Department of Pharmacology, Amiens-Picardie University Hospital, F-80054 Amiens, France

**Keywords:** animal model, chronic kidney disease, cardiovascular complications, calcified aortic valve disease

## Abstract

**Introduction.** This study addressed the hypothesis that subtotal nephrectomy associated with a high-phosphorus diet (5/6Nx + P) in rats represents a suitable animal model to mimic the cardiovascular consequences of chronic kidney disease (CKD) including calcified aortic valve disease (CAVD). Indeed, the latter contributes to the high morbidity and mortality of CKD patients and sorely lacks preclinical models for pathophysiological and pharmacological studies. **Methods.** Renal and cardiovascular function and structure were compared between sham-operated and 5/6 Nx rats + P 10 to 12 weeks after surgery. **Results.** As expected, 11 weeks after surgery, 5/6Nx + P rats developed CKD as demonstrated by their increase in plasma creatinine and urea nitrogen and decrease in glomerular filtration rate, estimated by using fluorescein-isothiocyanate-labelled sinistrin, anemia, polyuria, and polydipsia compared to sham-operated animals on a normal-phosphorus diet. At the vascular level, 5/6Nx + P rats had an increase in the calcium content of the aorta; a decrease in mesenteric artery dilatation in response to a stepwise increase in flow, illustrating the vascular dysfunction; and an increase in blood pressure. Moreover, immunohistology showed a marked deposition of hydroxyapatite crystals in the aortic valve of 5/6Nx + P rats. Echocardiography demonstrated that this was associated with a decrease in aortic valve cusp separation and an increase in aortic valve mean pressure gradient and in peak aortic valve velocity. Left-ventricular diastolic and systolic dysfunction as well as fibrosis were also present in 5/6Nx + P rats. **Conclusion.** This study demonstrates that 5/6Nx + P recapitulates the cardiovascular consequences observed in humans with CKD. In particular, the initiation of CAVD was shown, highlighting the interest of this animal model to study the mechanisms involved in the development of aortic stenosis and test new therapeutic strategies at an early stage of the disease.

## 1. Introduction

Despite major therapeutic advances, cardiovascular morbidity and mortality remain high in patients with chronic kidney disease (CKD) mainly due to the deleterious effects of uremic toxins on the cardiovascular system [1,2]. Among their effects, the potentiation of the development of ectopic calcification in arteries but mostly in cardiac valves remain poorly studied due in particular to the lack of representative animal models [2,3,4]. Calcified aortic valve disease (CAVD) is a slow and progressive disorder that ranges from mild valve thickening without obstruction of blood flow, termed aortic sclerosis, to severe calcification with impaired leaflet motion that characterize aortic stenosis. CAVD is highly prevalent and aortic stenosis progresses more rapidly in CKD patients than in the general population, contributing to increase cardiac afterload that further aggravates cardiac remodeling and dysfunction and significantly reduces survival [3,4]. To date, the mechanisms involved in the development of CAVD are poorly understood, and no specific therapeutic strategies are available. This is notably due to the absence of adequate animal models that truly mimic human AS [5,6]. Recently, it was proposed that the induction of CKD using subtotal nephrectomy associated with a high-phosphorus diet (5/6Nx + P) can promote aortic valve calcification, but the associated modifications in cardiovascular structure and function have to be evaluated [7].

In this context, the aim of the present study was to perform a comprehensive cardiovascular phenotyping of 5/6Nx + P rats with particular emphasis on the development of CAVD.

## 2. Methods

### 2.1. Animals and Experimental Procedures

All procedures were performed in accordance with the standards and ethical rules (CENOMEXA #24107). Twenty-four 10-week-old male Sprague–Dawley rats were purchased from Janvier Labs (Le Genest-Saint-Isle, France) and were aged until 18 weeks old, weighing 500–600 g. Then, 16 rats were submitted to a two-step surgical procedure performed by a single trained experienced operator (H.M.) in order to ensure reproducibility. The first step of the surgical procedure consisted of ligation of the upper branch of the left kidney artery, followed by a cauterization of the lower pole of the left kidney, leading to 2/3 of a non-functioning left kidney. One week later, the right kidney was removed, inducing 5/6 Nx. Then, 5/6 Nx rats were fed ad libitum a normal-calcium, high-phosphate diet (pellets containing 1% total calcium, 1.8% total phosphorus, Safe, Augy, France) until animal sacrifice 11 weeks after surgery. A group of 8 sham-operated rats (surgical laparotomy) eating a standard normal-calcium, normal-phosphate diet (pellets containing 1% total calcium, 0.9% total phosphorus, Safe) served as controls.

### 2.2. Blood and Renal Evaluations

At sacrifice, plasma blood samples were collected to assay creatinine, urea nitrogen, sodium, potassium, calcium, and phosphorus on Cobas^®^ analyzer (Roche, Mannheim Germany), as well as white and red blood cell counts, hemoglobin, and hematocrit levels. Uremic toxins including indoxyl sulfate, paracresyl sulfate, indole-acetic acid, trimethylamine oxide, and hippuric acid were assayed in the serum using a liquid chromatography–tandem mass spectrometry method as previously described [8]. Next, 24 h urine was collected 8 weeks after surgery using metabolic cages, allowing for the quantification of sodium, potassium, calcium, and phosphorus excretion, as well as albuminuria and creatininuria using the Catalyst Analyzer (IDEXX, Westbrook, ME, USA). Transcutaneous determination of glomerular filtration rate was performed 8 weeks after surgery by using fluorescein-isothiocyanate-labelled sinistrin as previously described [9].

### 2.3. Vascular Evaluations

Non-invasive measurements of systolic blood pressure were performed by tail cuff plethysmography (CODA, Kent Scientific Corporation, Torrington, CT, USA) 10 weeks after surgery. These measurements were performed in conscious and trained mice and consisted of two series of 10 cycles of measurements for each animal. At sacrifice, endothelium-dependent flow-mediated dilation was assessed on the second mesenteric-resistance artery segment. Briefly, the mesentery was removed and placed in cold oxygenated Krebs buffer. A 2–3 mm segment of third mesenteric resistance artery segment was isolated and mounted on an arteriograph (DMT, Aarhus, Denmark). Vessels were pre-constricted using 10^−5^ M phenylephrine before assessing the dilatory response to stepwise increase in intraluminal flow (0, 5, 10, 25, 50, 100, and 150 μL/min). Endothelium-independent dilatation to sodium nitroprusside (10^−5^ M) was assessed in preconstricted vessels. In addition, the thoracic aorta was removed and cut in half. One segment was washed once with PBS and then decalcified with 0.6 N HCl overnight at 4 °C. The calcium content in the HCl supernatant was colorimetrically analyzed by the o-cresolphthalein complexone method [10]. Calcium content in aortic rings were corrected by aortic dry weight with aortas dried overnight at 37 °C. In addition, calcium deposition was evaluated on the second thoracic segment using 7 μm thick histological slices stained with Alizarin red [11].

### 2.4. Cardiac Evaluations

For transthoracic echocardiography, rats were anesthetized with isoflurane 2% in 21% oxygen/compressed air at 1 L/min) and placed on a heated plate to maintain body temperature at 37.5 °C, the chest shaved, and a Vivid 7 ultrasound echograph (GE Healthcare, Buc, France) equipped with a M12L linear probe operating at 14 MHz and fitted out with Echopac PC software (GE Medical Systems) was used [10].

#### 2.4.1. Left-Ventricular Systolic Function

Briefly, a two-dimensional parasternal long-axis view of the left ventricle was obtained at the level of the papillary muscle, in order to record M-mode tracings. Left-ventricular (LV) end-diastolic (EDD) and systolic diameters (ESD) and end-diastolic anterior and posterior LV wall thicknesses were measured by the American Society of Echocardiology leading-edge method from at least 3 consecutive cardiac cycles. LV fractional shortening (FS) was calculated from the variation in LV diameters as FS (%) = ((LVEDD − LVESD)/LVEDD) × 100, and the LV ejection fraction (EF) was calculated by the Teicholz formula from LV diameters. Cardiac output (CO) was calculated by multiplying the stroke volume (the LV end-diastolic volume minus LV end-systolic volume) by the heart rate.

#### 2.4.2. Left-Ventricular Diastolic Function

Doppler measurements were made at the tip of the mitral leaflets for diastolic filling profiles in the apical four-chamber view, allowing us to determine the E/e’ ratio (mitral inflow E wave/e’ tissue Doppler mitral annulus velocity) as an estimate of diastolic function.

#### 2.4.3. Aortic Valve Assessment

In addition, the aortic valve peak flow velocity and aortic valve mean gradient were measured in the apical five-chamber view by continuous wave doppler in systole. The presence or absence of aortic regurgitation was visualized in this five-cavity view by color Doppler. Furthermore, aortic stenosis was estimated in two-dimensional parasternal long-axis view by measuring the aortic valve cusp separation in systole.

At sacrifice, the heart was harvested and weighed, and a section of the left ventricle was snap-frozen for subsequent determination of LV fibrosis, using 8 µm thick histological slices stained with Sirius Red as previously described [12]. In addition, the aortic valve was carefully dissected and incubated over 24 h at 4 °C in a solution of a 20 nM Osteosense 680Ex^®^ (Perkin-Elmer, Waltham, MS, USA) in PBS. This fluorescent probe binds to hydroxyapatite with high affinity and thus allows for the detection of microcalcifications. Then, the valve was rinsed and snap-frozen for subsequent analysis using 8 µm thick histological slices with mounting medium containing DAPI. Pictures were acquired on an epifluorescence microscope (Axio Imager 1, Zeiss, Jena, Germany) equipped with an apotome using the Cyanine 5.5 filter for calcification detection and a DAPI filter for cell nuclei detection.

### 2.5. Statistics

All data are presented as mean ± SEM unless differentially indicated. Data normality was verified in sham-operated and 5/6Nx + P groups using the Shapiro–Wilk test. Comparisons between groups were performed using Student’s t-test for normally distributed data and using the Wilcoxon rank-sum test for non-normally distributed data. The comparison between groups for endothelium-dependent relaxations to stepwise increase in flow was performed using repeated measures ANOVA.

## 3. Results

**Figure 1:**
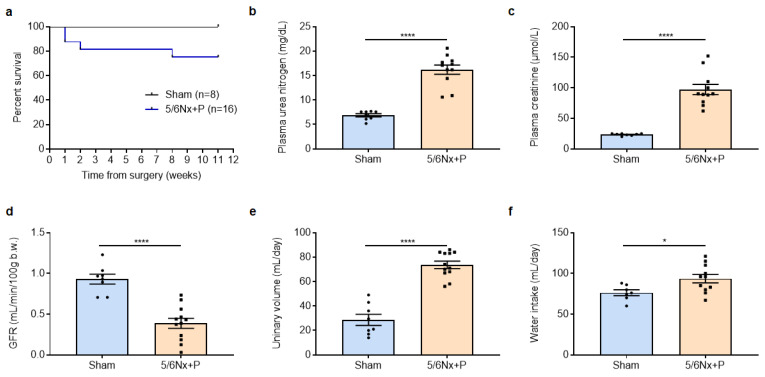
Eleven-week survival after surgery (**a**), plasma creatinemia (**b**), urea nitrogen (**c**), glomerular filtration rate (GFR, **d**), urinary volume (**e**), and water intake (**f**) measured 10 to 11 weeks after surgery in sham-operated and 5/6 nephrectomized rats receiving a high-phosphate diet (5/6Nx + P). * *p* < 0.05, **** *p* < 0.0001.

The mortality rate was 25% in the 5/6Nx + P group during the 11-week follow-up, while no death was observed in the sham-operated group (shown in Figure 1a). First of all, 5/6Nx + P rats developed renal failure, as shown by the increase in plasma urea and creatinine and the reduction of glomerular filtration rate compared to sham-operated rats (shown in Figure 1b–d). In addition, 5/6Nx + P rats had polyuria and polydipsia (shown in Figure 1e,f). Biochemical and hematological analyses are shown in Table 1. As expected from the experimental diet, phosphaturia was dramatically increased in 5/6Nx + P rats. Urinary calcium, sodium, and potassium levels were also all increased in 5/6Nx + P rats. However, the plasma levels of calcium and phosphorus as well as the calcium-phosphorus product remained similar between groups. In addition, the plasma sodium level was unchanged, but hyperkalemia was observed in 5/6Nx + P rats as well as hypoproteinemia. Furthermore, anemia developed in 5/6Nx + P rats as shown by the decrease in the number of blood erythrocytes and in hemoglobin and hematocrit levels without change in leukocyte number. As shown in Table 2, the concentrations of uremic toxins were also increased in the serum of 5/6Nx + P rats compared to sham-operated rats.

**Figure 2:**
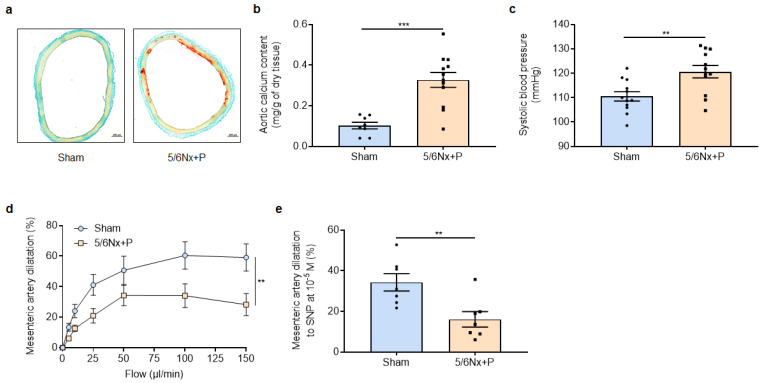
Representative images (**a**) and relative values of calcium content (**b**) of aortic rings stained with Alizarin red, systolic blood pressure (**c**), mesenteric artery endothelium-dependent dilatation to stepwise increase in flow (**d**), and endothelium-independent dilatation to 10^−5^ M sodium nitroprusside (**e**) obtained 10 to 11 weeks after surgery in sham-operated and 5/6 nephrectomized rats receiving a high-phosphate diet (5/6Nx + P). ** *p* <0.01, *** *p* < 0.001.

At the vascular level, 5/6Nx + P rats displayed aortic calcification, as shown by the increase in aortic calcium content and Alizarin red staining compared to sham-operated rats (Figure 2a,b). This was associated with the presence of systemic hypertension as well as vascular dysfunction, as shown by the impairment of both mesenteric artery endothelium-dependent and -independent dilatation (shown in Figure 2c–e).

**Figure 3:**
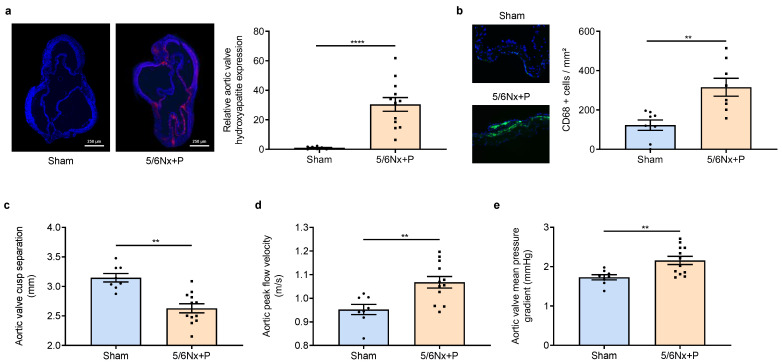
Representative images and relative values of hydroxyapatite expression of aortic valves stained with Osteosense 680Ex^®^ (**a**), representative images and values of CD68+ cells per mm² (green and blue staining represent CD68+ cell and DAPI nuclear marker, respectively) (**b**), aortic valve cusp separation (**c**), peak flow velocity (**d**), and mean pressure gradient (**e**) obtained 10 to 11 weeks after surgery in sham-operated and 5/6 nephrectomized rats receiving a high-phosphate diet (5/6Nx + P). ** *p* < 0.01, **** *p* < 0.0001.

**Figure 4:**
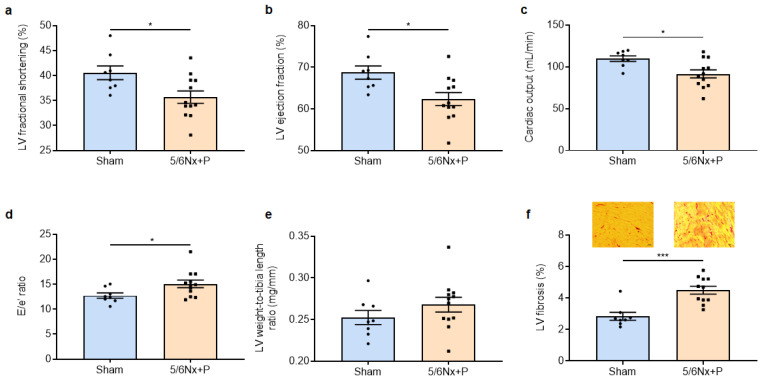
Left-ventricular (LV) fractional shortening (**a**), ejection fraction (**b**), cardiac output (**c**), E/e’ ratio (mitral inflow E wave/e’ tissue Doppler mitral annulus velocity) (**d**), LV weight-to-tibia length ratio (**e**), and representative images of Sirius red-stained LV sections and level of fibrosis (**f**) obtained 10 to 11 weeks after surgery in sham-operated and 5/6 nephrectomized rats receiving a high-phosphate diet (5/6Nx + P). * *p* < 0.05, *** *p* < 0.001.

Of major importance, immunohistological examination of aortic valve sections allowed us to demonstrate the presence of hydroxyapatite crystals in 5/6Nx + P rats but not in sham-operated rats (shown in Figure 3a). In addition, there was an increased CD68+ cells in aortic valves of 5/6Nx + P rats (shown in Figure 3b), demonstrating macrophage infiltration and inflammation. Echocardiography showed classical structural and hemodynamical changes associated with aortic valve calcification, i.e., a decrease in aortic valve cusp separation and an increase in aortic valve peak flow velocity and aortic valve mean pressure gradient (shown in Figure 3c–e). In addition, alterations in cardiac systolic and diastolic function were observed with a slight but significant decrease in LV fractional shortening, ejection fraction, and cardiac output, and an increase in the E/e’ ratio (shown in Figure 4a–d), while cardiac remodeling was supported from the nonsignificant increase in LV weight and the presence of LV fibrosis (shown in Figure 4e,f).

## 4. Discussion

The major finding of this study is that the induction of CKD by subtotal nephrectomy in rats combined with a high-phosphate diet allowed us to promote the development of aortic valvular calcification with classical structural and hemodynamical repercussions of CAVD.

At this time, no reliable animal model for human CAVD exists, restraining the determination of the mechanisms involved in the development of aortic stenosis and the discovery of adequate therapeutic strategies. Only swine has been shown to spontaneously develop CAVD, while rabbit or murine models require genetic and/or external interventions, are time-consuming, and only a small proportion of the animals truly develop CAVD [5,6]. For this study, we decided to focus on a rat model of subtotal nephrectomy associated with a high-phosphate diet, since CKD remains a major risk factor for the development of aortic stenosis [3,4], and because one recent work demonstrates that it is associated with the development of valvular calcification [7]. We chose a phosphate diet containing 1.8% phosphorus that allowed for maintaining all other dietary nutrients at the same values than the normal 1% phosphate diet and a duration of 10 to 11 weeks since a 8-week period appeared to not be sufficient [7].

As expected from this model, 5/6Nx + P rats quickly developed all the hallmarks of CKD with decreased kidney function, a uremic state, and anemia. In this context, and despite the absence of change in the plasma calcium-phosphorus product, 5/6Nx + P rats displayed ectopic calcification at the arterial level and in the aortic valve. The latter calcifications were highlighted thanks to the use of a synthetic fluorescent bisphosphonate, which binds to hydroxyapatite crystals with high affinity [12], thus allowing us to confirm previous results obtained using sophisticated methods based on electron microscopy and X-ray diffraction [7].

As suggested in CKD patients, vascular calcification may contribute to the development of hypertension and vascular dysfunction in 5/6Nx + P rats. Importantly, we can demonstrate for the first time using echocardiography that the aortic valvular calcification and inflammation is associated with a significant decrease in the aortic valve cusp separation and an increase in aortic valve peak flow velocity and aortic valve mean pressure gradient, which are classically used to categorize patients with CAVD. Although it is not possible to distinguish between the effects of renal dysfunction/uremic toxins and a putative early consequence of CAVD, cardiac diastolic and systolic functions were slightly altered, and cardiac remodeling was already present in 5/6Nx + P rats. The alteration in systolic function was not previously observed in this model by Wang et al. [7], but it should be kept in mind that we used older animals (18-week-old vs. 8-week-old rats) and that the severity of 5/6Nx and thus of kidney damage may vary notably depending on the extent of kidney cauterization. In our 5/6Nx + P rats, the left-ventricular remodeling (as shown by the increase in left ventricle weight and fibrosis) and both the systolic and diastolic dysfunctions of the left ventricle (as shown by the decrease in fractional shortening and the increase in the E/e’ ratio, respectively) were consistent with type 4 cardiorenal syndrome [13]. This uremic cardiopathy was associated with an increase in afterload objectified in vivo by the slight but significant increase in arterial blood pressure and ex vivo by the impaired endothelial function. This led to the reduction in cardiac performance (as shown by the decrease in ejection fraction and cardiac output) that will aggravate until heart failure development.

This study demonstrates that subtotal nephrectomy combined with a high-phosphorus diet in SD rats allows us to recapitulate, over a short period, the cardiovascular hallmarks of CKD including CAVD and its hemodynamic consequences. A longer follow-up of the 5/6Nx rats on this high-phosphorus diet may be helpful to potentialize the development of aortic stenosis and cardiac alterations. However, this model already represents a promising experimental tool to study the pathophysiology of CAVD and test the impact of new therapeutic strategies targeting the valvular calcification process at an early stage, in the expected way of finally reversing the course of the disease, improving patients’ health, and limiting the need for aortic valve replacement.

## Figures and Tables

**Table 1:** Hematological and biochemical analyses, 9 to 11 weeks after surgery, in sham-operated rats and 5/6 nephrectomized rats receiving a high-phosphate diet (5/6Nx + P).

Parameters	Sham n = 8	5/6Nx + P n = 11 or 12	*p*-Value
***Blood hematological parameters (at 11 weeks post-surgery)***			
Leukocytes, × 10^9^/L	6.51 ± 0.46	5.78 ± 0.53	0.3655
Erythrocytes, × 10^12^/L	9.2 ± 0.19	6.09 ± 0.41	<0.0001
Hematocrit, %	0.49 ± 0.01	0.35 ± 0.02	0.0006
Hemoglobin, g/dL	14.99 ± 0.32	10.63 ± 0.69	0.0003
***Plasma biochemical parameters (at 11 weeks post-surgery)***			
Sodium, mmol/L	142.3 ± 0.7	142.5 ± 1.1	0.8855
Potassium, mmol/L	3.87 ± 0.28	4.49 ± 0.12	0.0399
Calcium, mmol/L	2.42 ± 0.07	2.25 ± 0.09	0.1940
Phosphorus, mmol/L	1.72 ± 0.08	2.06 ± 0.29	0.3407
Calcium-phosphorus product	4.15 ± 0.18	4.41 ± 0.44	0.6417
Proteins, g/dL	5.53 ± 0.09	5.32 ± 0.05	0.0377
***24 h urine biochemical parameters (at 9 weeks post-surgery)***			
Creatinine, mmol/L	7.05 ± 0.83	2.53 ± 0.18	<0.0001
Sodium-to-creatinine ratio	7.4 ± 0.34	25.6 ± 1.27	<0.0001
Potassium-to-creatinine ratio	30.4 ± 0.6	60.5 ± 1.9	<0.0001
Calcium-to-creatinine ratio	0.11 ± 0.01	0.30 ± 0.03	0.0005
Phosphorus-to-creatinine ratio	0.45 ± 0.14	30.71 ± 1.18	<0.0001
Albumin-to-creatinine ratio, mg/mmol	275 ± 26	583 ± 105	0.011

Data are mean ± SEM.

**Table 2:** Serum concentration of uremic toxins assayed 11 weeks after surgery in sham-operated rats and 5/6 nephrectomized rats receiving a high-phosphate diet (5/6Nx + P).

	Sham n = 8	5/6Nx + P n = 12	*p*-Value
Indoxyl sulfate (mg/L)	0.23 ± 0.05	10.73 ± 1.98	0.0004
Paracresyl sulfate (mg/L)	0.04 ± 0.01	8.23 ± 1.19	<0.0001
Indole acetic acid (mg/L)	0.18 ± 0.02	0.45 ± 0.05	0.0002
Triethylamine oxide (mg/L)	0.11 ± 0.02	1.65 ± 0.33	0.016
Hippuric acid (mg/L)	2.11 ± 0.16	8.23 ± 1.19	0.0006

Data are mean ± SEM.

## Data Availability

Data are available from the corresponding author upon reasonable request.
